# The importance of tumor microenvironment modulations in the progression of pancreatic intraductal papillary mucinous neoplasms^†^

**DOI:** 10.1002/path.6460

**Published:** 2025-07-30

**Authors:** Antonio Pea, Claudio Luchini

**Affiliations:** ^1^ Department of General and Pancreatic Surgery ‐ The Pancreas Institute Verona University Hospital Trust Verona Italy; ^2^ Department of Diagnostics and Public Health, Section of Pathology University of Verona Verona Italy; ^3^ ARC‐Net Research Center University of Verona Verona Italy

**Keywords:** tumor microenvironment, immune evasion, intraductal papillary mucinous neoplasm, pancreatic ductal adenocarcinoma

## Abstract

Intraductal papillary mucinous neoplasms (IPMNs) of the pancreas have attracted substantial attention since they represent the most prevalent macroscopic precursor of pancreatic cancer. Most lesions show an epithelium with low‐grade dysplasia and will remain indolent and unknown to the patient. Notably, a subgroup of IPMNs will progress to invasive cancer through a stepwise process characterized by the accumulation of specific genomic alterations and concomitant modifications of the tumor microenvironment (TME). The manuscript of Jamouss *et al*, recently published in *The Journal of Pathology*, expands the current knowledge on TME dynamics in IPMNs. The neoplastic progression of IPMNs is paralleled by a shift toward an immunosuppressive TME, with depletion of cytotoxic T cells, elevated expression of immune checkpoint molecules, including PD‐L1 and VISTA, and increased density of macrophages. Overall, TME modifications are crucial in the progression of pancreatic IPMNs, calling for potential therapeutic strategies focused on TME modulations for cancer interception. © 2025 The Author(s). *The Journal of Pathology* published by John Wiley & Sons Ltd on behalf of The Pathological Society of Great Britain and Ireland.

## Commentary

The study of precursors to pancreatic ductal adenocarcinoma (PDAC) represents a critical opportunity for investigating the complex mechanisms involved in pancreatic oncogenesis. In this scenario, pancreatic intraepithelial neoplasia (PanIN) and intraductal papillary mucinous neoplasm (IPMN) are the most extensively studied precancerous lesions of PDAC. A wealth of literature has elucidated the genomic alterations that drive malignant transformation in these precursors. More recently, a growing research focus has been tumor microenvironment (TME) modifications and their contribution to pancreatic oncogenesis in both PanINs and IPMNs.

**Figure 1:**
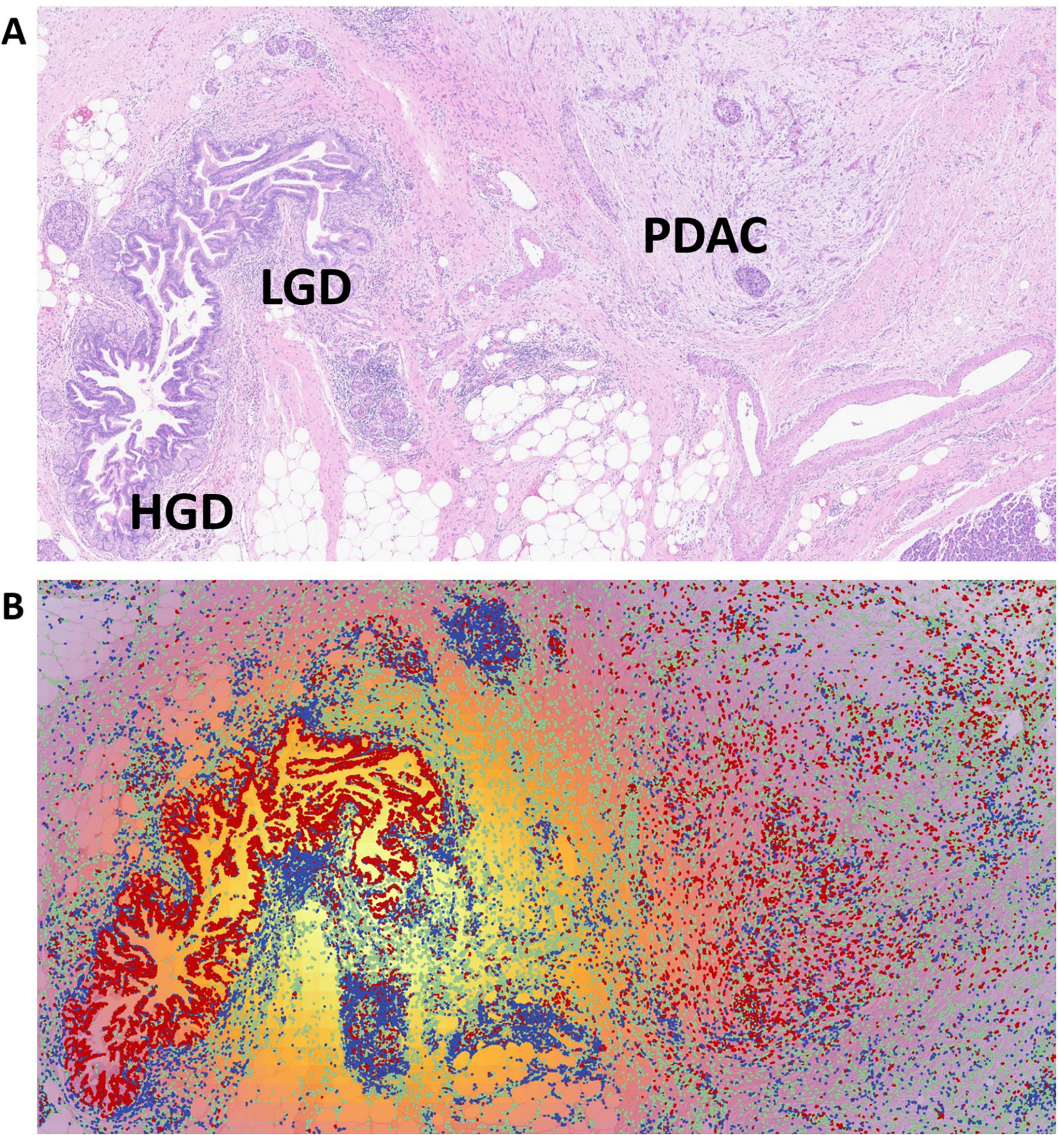
Representative figure of modifications of TME in progression of pancreatic IPMNs. (A) IPMN with low‐grade dysplasia (LGD) and high‐grade dysplasia (HGD) and an associated invasive adenocarcinoma (PDAC) (hematoxylin–eosin, original magnification ×10). (B) Same slide analyzed with digital pathology (using QuPath open‐source software platform, version 0.2.3), where a random tree forest classifier was trained to classify cells into epithelial (red), stromal (green), and lymphocytes (blue). The density map for lymphocytes evidenced their higher density in the region of IPMN with LGD compared to the regions with HGD and with PDAC. This is one of the key concepts in the TME modifications during the progression of IPMN to invasive cancer.

PanINs are microscopic precursors and show an immunosuppressive TME, which includes myeloid‐derived suppressor cells, regulatory T cells, and tumor‐associated macrophages (TAMs) [1]. A complex interplay between regulatory T cells and other immune cells in the TME, such as CD11c^+^ dendritic cells, results in the depletion of cytotoxic CD8^+^ T cells, supporting PanIN progression to invasive cancer [2]. Regulatory T cells are found in similar numbers and concentrations in PanINs and IPMNs, which are macroscopic, cystic precursors to PDAC [2]. However, unlike PanINs, IPMNs show a different and variable population of cytotoxic CD8^+^ T lymphocytes. Specifically, IPMNs show a higher proportion of cytotoxic CD8^+^ T cells in lesions with low‐grade dysplasia than in lesions with high‐grade dysplasia [3]. IPMNs with low‐grade dysplasia also have activated CD4^+^ T lymphocytes that persist in the shift from low‐ to high‐grade dysplasia [3]. While progressing from IPMNs with low‐grade dysplasia to IPMNs with high‐grade dysplasia and, ultimately, to invasive carcinoma, the TME shifts toward an immunosuppressive state (Figure 1) [2, 3]. Indeed, recent single‐cell and spatial transcriptomics studies demonstrate that regions of high‐grade dysplasia are relatively depleted of T cells and show a trend toward macrophage enrichment compared to regions of low‐grade dysplasia [3, 4]. It is now clear that the TME is a dynamic environment that evolves in parallel with tumor progression. However, a precise understanding of TME composition during various phases of pancreatic oncogenesis is still lacking.

The investigation by Jamouss *et al*, published recently in the *The Journal of Pathology*, brings new and fascinating insights to this topic and expands our knowledge of TME dynamics in IPMNs [5]. The authors quantitatively characterized the TME composition by analyzing different types of immune cell and immune checkpoint molecules in two different cohorts of pancreatic IPMN, identifying specific modifications in the TME composition associated with tumor progression and clinical behavior [5]. The first cohort of samples was composed of tissue microarrays from 99 patients who underwent surgical resection for IPMN. Of those, 52 patients had only low‐grade dysplasia, 17 had only high‐grade dysplasia, and 30 had separately assessed regions with low‐grade and high‐grade dysplasia. The second cohort was composed of an additional, independent cohort of 88 specimens from surgically resected pancreata of patients with noninvasive IPMN, already assessed in a prior study that looked at the risk of recurrence after surgery [6]. The current analysis was performed on samples from both cohorts with chromogenic immunohistochemistry coupled with digital pathology to quantify the density of the different types of immune cells. In the first cohort, neoplastic epithelium was annotated to correlate immune cell densities with the grade of IPMN dysplasia. In the second cohort, to assess recurrence risk and clinical outcomes, all neoplastic epithelium was annotated regardless of the grade of IPMN dysplasia. For the first cohort, several immunological and checkpoint markers were analyzed, including CD45, CD3, CD4, CD8, CD20, CD68, CD163, LAG3, V‐domain immunoglobulin suppressor of T‐cell activation (VISTA), FOXP3, TIM3, and PD‐L1, along with different types of mucins for better identifying the histological subtype (i.e., gastric, pancreatobiliary, intestinal). For the second cohort, the specimens were stained for the immune checkpoint markers TIM3, VISTA, and PD‐L1.

The authors showed that IPMN displayed a clear progression to a more immunosuppressive TME in the transition from low‐grade to high‐grade dysplasia. Indeed, IPMNs with high‐grade dysplasia were characterized by elevated expression of immune checkpoint molecules (including PD‐L1 and VISTA), increased macrophage density, and decreased cytotoxic CD8^+^ T cells. While the modifications affecting the macrophage population were limited to focal regions of high‐grade dysplasia, T‐cell alterations affected the entire IPMN. Importantly, by analyzing IPMNs that displayed both high and low grades of dysplasia within the same sample, the authors showed reduced overall T cells and cytotoxic T‐cell densities in patients with IPMNs with both grades of dysplasia compared with those who had only low‐grade dysplasia. This finding suggests that the presence of focal high‐grade dysplasia is associated with changes in T‐cell densities that involve the entire IPMN. This specific finding corroborates the results of a recent study by Hernandez *et al*, in which the authors identified those regions, defining them as ‘progressed’ low‐grade dysplasia [7].

The authors also analyzed macrophage composition in the TME of lesions with varying grades of dysplasia. They used CD68, a pan‐macrophage marker, and CD163, a marker of tumor‐associated macrophage class 2 (TAM‐2), which are considered tumor‐promoting due to their ability to facilitate tumor progression. The authors reported elevated densities of CD68^+^ and CD163^+^ macrophages in IPMN regions with high‐grade dysplasia. However, the densities of these populations in the adjacent IPMN regions with low‐grade dysplasia were variable. The elevation of CD163^+^ macrophages in regions with high‐grade dysplasia confirms the role of TAM‐2 as tumor‐promoting immune cells also in IPMNs. Such macrophages, also secreting immunosuppressive cytokines and chemokines, including IL‐10, IL‐13, and TGF‐β, contribute to immunosuppression and tumor progression and have also been associated with poor prognosis in patients with PDAC [2]. For the first time, Jamouss *et al* also showed that the increase of CD68^+^ and CD163^+^ macrophages was also matched with an increase in PD‐L1 and VISTA, which are novel targetable immune checkpoints.

Interestingly, Jamouss *et al* found that VISTA was associated with poorer clinical outcomes after IPMN resection. VISTA has been noted to be highly expressed in PDAC compared with the normal pancreas. Furthermore, mechanistic studies *in vitro* in co‐culture models suggest that VISTA impacts cytokine production by CD3^+^/CD8^+^ T cells, favoring the creation of an immunosuppressive environment [8]. Of note, the study by Jamouss *et al* also highlighted the potential clinical importance of VISTA in IPMNs and, possibly, in IPMN‐derived cancers. However, it is important to acknowledge that a previous investigation reported no differences in terms of overall survival between higher and lower VISTA expression in IPMN‐associated invasive cancers [9]. The presence of conflicting results on this topic underscores the urgent need for further studies on TME dynamics in pancreatic oncogenesis, also elaborating standardized approaches to biomarker staining and scoring.

The study of TME modifications during the transition between low‐grade and high‐grade dysplasia in IPMNs represents a unique opportunity for better understanding pancreatic carcinogenesis and, ultimately, PDAC biology. The study by Jamouss *et al*, which is based on the largest sample size of TME‐characterized IPMNs in the literature, is an important addition to our knowledge of TME dynamics that accompany IPMN progression. The authors provide detailed evidence of a clear shift in the IPMN TME toward immune evasion that parallels the transition from low‐grade to high‐grade dysplasia observed in neoplastic cells. These data, which were validated in two independent cohorts, provide a rational basis for designing potential interception strategies in pancreatic oncogenesis, targeting immune features of the TME. Functional studies clarifying the role of immune cell types in the TME will also be important. While Jamouss *et al* elegantly showed that CD8^+^ T cells were enriched in the TME of IPMNs with low‐grade dysplasia, it remains to be determined whether these cells are capable of tumor antigen recognition and are able to generate antitumor activity. Similarly, it is unclear whether treatment with checkpoint inhibitors will result in clinically meaningful responses. Although responses in patients with advanced PDAC treated with anti PD‐L1 or anti CTLA‐4 antibodies have been so far limited [10], the data by Jamouss *et al* would suggest a role for checkpoint inhibitors in noninvasive IPMN as a chemoprevention strategy that warrants further investigation.

## Author contributions statement

AP and CL provided study conception and design, discussion of the topic, writing of the manuscript and final editing and approval of the present version.

## Data Availability

Data sharing not applicable to this article as no datasets were generated or analyzed during this study.
